# Fluoxetine Exposure During Sexual Development Disrupts the Stress Axis and Results in Sex- and Time- Dependent Effects on the Exploratory Behavior in Adult Zebrafish *Danio rerio*

**DOI:** 10.3389/fnins.2019.01015

**Published:** 2019-09-19

**Authors:** Marilyn N. Vera-Chang, Antony D. St-Jacques, Chunyu Lu, Thomas W. Moon, Vance L. Trudeau

**Affiliations:** ^1^Department of Biology, University of Ottawa, Ottawa, ON, Canada; ^2^Department of Chemistry and Biomolecular Sciences, Centre for Catalysis Research and Innovation, University of Ottawa, Ottawa, ON, Canada

**Keywords:** fluoxetine, cortisol, behavior, sexual development, zebrafish

## Abstract

The antidepressant fluoxetine (FLX), generally the first line of pharmacological treatment in adolescents and pregnant women with affective disorders, is an emerging endocrine disruptor that is also released to the environment through sewage. Recently, we demonstrated that FLX exposure during the first 6 days of life in zebrafish (ZF; *Danio rerio*) induced a male-specific reduction in the exploratory behavior in the adult ZF that was linked to a reduction in cortisol production that persisted across three generations. Here we investigated sex differences in the behavioral and stress responses following FLX (0.54 and 54 μg^⋅^L^–1^) exposure during two periods of sexual development in ZF; early (0–15 days post-fertilization, dpf) and late (15–42 dpf). Our findings revealed that the stress response in females was reduced compared to that of males independent of the treatment. We also found that FLX reduced total body cortisol levels in the adult ZF regardless of sex and window of exposure. The hypocortisol phenotype of our FLX-treated fish was associated with behavioral alterations in the adult fish, which depended on the window of exposure; males were more sensitive to FLX during early development whereas females were affected during late development. A sexually dimorphic behavioral response induced by the low cortisol phenotype was observed in the FLX-treated ZF; females had higher exploratory activity whereas the males had reduced behavior. In conclusion, FLX results in sex- and window of exposure-specific effects on the behavioral activities in adult ZF. These findings highlight the importance of sex differences and timing on the long-term effects of antidepressant treatments. Knowledge of the sex-specific effects of antidepressants and the importance of early life exposure to chemical stressors may help us understand the impact of highly prescribed drugs such as FLX on the fetus from FLX-treated pregnant women as well as aquatic species in environments receiving sewage effluents.

## Introduction

Stress, including those caused by therapeutic drugs during sensitive periods of development, can permanently shape the adult neuroendocrine and behavioral responses to internal and external cues in humans, rodents, and teleost fish (Koehl et al., 2000; Bale et al., 2010; Pawluski et al., 2012; Wolstenholme et al., 2012, 2013; Aluru et al., 2017). A major chemical stressor with emerging concerns because of its endocrine disruptive properties is fluoxetine (FLX), the active ingredient in well-known antidepressants including Prozac. Fluoxetine, a selective serotonin [5-hydroxytryptamine (5-HT)] reuptake inhibitor (SSRI), is generally the first line of pharmacological treatment for affective disorders in adolescents and in women during pregnancy and the postpartum period (Latendresse et al., 2017; Locher et al., 2017; Man et al., 2017; Morkem et al., 2017; Sarginson et al., 2017). Given that SSRIs are transferred from the treated mother to the fetus across the placenta (Hendrick et al., 2003; Rampono et al., 2004; Kim et al., 2006) and that they are excreted in the breast milk (Kim et al., 2006), concerns have been raised regarding exposure to this chemical during developmentally sensitive windows including the prenatal period and puberty. Additionally, the continuous discharge and improper disposal into wastewater streams have led to the detection of FLX in aquatic environments at concentrations as high as 929 ng^⋅^L^–1^ (Bueno et al., 2007), rendering aquatic organisms such as fish susceptible to the endocrine-disrupting effects of FLX.

The SSRIs exert their therapeutic actions by enhancing serotonergic neurotransmission through inhibition of 5-HT reuptake transporters on presynaptic neurons (Wong et al., 1995). Exposure to SSRIs can upset 5-HT-mediated processes in the exposed individual, including but not limited to its prominent role in the programming of the hypothalamic–pituitary–adrenal (HPA) axis in mammals (Andrews and Matthews, 2004; Oberlander, 2012), which is highly plastic during development (Loman et al., 2010; Heim and Binder, 2012). Egan et al. (2009) were the first to show that a high dose of fluoxetine (100 μg^⋅^L^–1^, for 2 weeks) decreased whole-body cortisol levels in adult zebrafish (ZF, *Danio rerio*). Recently, we reported that exposure to FLX from 0 to 6 days post-fertilization (dpf) in ZF disrupts the hypothalamic-pituitary-interrenal (HPI) axis (Vera-Chang et al., 2018), homologous to the HPA axis in mammals. The early life exposure to FLX suppressed their adult stress-induced cortisol levels (Vera-Chang et al., 2018), the primary stress hormone in teleosts (Romero, 2004). This effect persisted for three consecutive generations in the unexposed descendants without diminution and was more pronounced in males (Vera-Chang et al., 2018). The impairment in cortisol production was linked to a male-specific decrease in the exploratory activity of the adult ZF (Vera-Chang et al., 2018). Although there is limited research on the effects of sexually dimorphic effects of FLX, some studies report on differences in the alteration of various behaviors following prenatal and early postnatal exposure to FLX in rodents. In mice, prenatal and early postnatal FLX treatment decreased ambulation in the open–field test at postnatal day (PND) 40, an effect observed solely in males (Lisboa et al., 2007). In the same study, the authors reported a female-specific effect on the forced swimming test (increased immobility time) at PND 30 and 70 following the same FLX treatment (Lisboa et al., 2007). In rats (at PND 27), a male-specific decrease in the time spent grooming a novel conspecific followed prenatal and postnatal treatment with FLX (Gemmel et al., 2017). In another study, prenatal and postnatal FLX treatment resulted in an increase in social investigation in adult female rats only, while in adult male rats, the same treatment increased social play (pouncing, nape attacks) (Gemmel et al., 2019). An increase in anxiety-like behaviors resulting from the elevated plus maze and the novelty suppressed feeding test were observed in the adult male offspring following FLX administration to lactating rat dams (Gobinath et al., 2016). It is known that the adaptive and/or maladaptive behavioral responses resulting from exposure to early life stressors are strongly correlated to the age of the exposed organism during which the stressor is experienced (Luine et al., 2007). These age-dependent effects are mainly driven by the levels of circulating sex steroids (i.e., androgens and estrogens) (McCormick et al., 2005) which through organizational and activational effects (Rosenfeld et al., 2017), play a critical role in developing sexual dimorphism within the brain (Frye et al., 2012). Thus, critically missing is the knowledge whether the sexual dimorphic behavioral alterations in adults induced by FLX is dependent on the window of exposure.

The limited data available that address sex differences and examine the disruption of the stress axis resulting from early life exposure to FLX have yielded mixed results. For instance, administration of FLX to lactating rat dams enhanced the stress-induced corticosterone levels, the primary stress hormone in rodents, in the adult male offspring (Gobinath et al., 2016). In another study, postnatal FLX treatment through lactation for 28 days decreased basal circulating corticosterone levels in adolescent male rats, but not female offspring (Pawluski et al., 2012). Even though in these studies, postnatal FLX treatment in rodents resulted in different directions of disruption in the stress hormone corticosterone, they are both in agreement with our work (Vera-Chang et al., 2018) regarding the increased susceptibility of males to disruption by early life exposure to FLX in ZF. The contradictory findings on the effects of corticosterone levels by maternal postpartum FLX treatment reported in the studies with rodents are likely due to methodological differences including timing and method of FLX administration (use of stressful methods). Additionally, the methodology conducted to develop their model of maternal postpartum stress/depression may also influence the effects of the exposure. Therefore, further research is warranted to determine the effects of FLX treatment during different developmental windows without the confounding effects of maternal stress and/or depression.

In this regard, the ZF is a particularly suitable model for stress research (Steenbergen et al., 2011) and for other human-related brain disorders (Howe et al., 2013; Kalueff et al., 2014). Fertilization and development are external, and thus the early embryonic environment can be easily controlled and manipulated. Here, we tested the hypothesis that exposure to FLX during two periods spanning sexual development (early and late) impairs the stress axis of adult ZF, consequently disrupting their behavioral responses in a sex- and time-dependent manner.

## Materials and Methods

### Experimental Animals and Exposure

**FIGURE 1:**
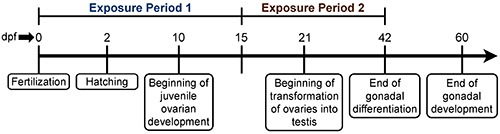
Timeline of the sex developmental period in ZF. Exposure 1 was performed from 0 to 15 dpf during early sexual development while exposure 2 was conducted from 15 to 42 dpf during the sex differentiation period (Orban et al., 2009; Luzio et al., 2015).

Adult ZF (AB strain) were obtained from Big Al’s Aquarium in Ottawa, ON, Canada and allowed to acclimate for 4 weeks in flow-through aquaria systems supplied with heated (28.5 ± 0.2°C), aerated, dechloraminated City of Ottawa tap water (hereby referred to as system water) on a 14 h light:10 h dark photoperiod prior to generating the embryos for this study. Sixteen breeding cages (Aquatic Habitats; Apopka, FL, United States) containing system water were set up in the late afternoon with a plastic divider separating the females from the male in a ratio of 2F:1M and left undisturbed until the separator was removed the following morning. Embryo collection was carried out as described in our previous study (Vera-Chang et al., 2018). Once collected, embryos from different spawning pairs were pooled to avoid batch effects and subsequently randomly divided into two groups: the first group was used for exposure period 1 (early sexual development) and the second group for exposure period 2 (late sexual development) (Figure 1). Sexual development in ZF is highly sensitive to sex steroids as sex determination, in addition to the differentiation and development of their gonads, are driven primarily by androgens and estrogens (Luzio et al., 2015), which are known to play a critical role in the sex-specific effects induced by exposure to stressors (Frye et al., 2012). Sexual development in ZF begins at about 10 dpf with the differentiation of the juvenile ovary in both sexes, regardless of the actual sexual genotype. At approximately 21 dpf, the juvenile ovaries in males driven by the actions of androgens undergo apoptosis and are transformed into testes whereas in females, the high levels of estrogens induce the continuation of ovary development. At ∼60 dpf, both gonads are completely developed (Figure 1; Uchida et al., 2004; Orban et al., 2009; Tong et al., 2010).

Embryos assigned to exposure period 1 were immediately distributed into glass Petri dishes containing either embryo medium alone (control; CTR) or supplemented with one of two concentrations of FLX (Millipore Sigma; Oakville, ON, Canada): 0.54 μg^⋅^L^–1^ (LOW) or 54 μg^⋅^L^–1^ (HIGH). These FLX concentrations were based on our previous study (Vera-Chang et al., 2018), which demonstrated a disruption of the stress axis and behavioral responses in adult ZF following a 6-day embryonic exposure. Concentrated stock solutions of FLX were prepared in the same water that was used for the embryo medium preparation. The FLX concentrations were measured to be 77.5 ± 9.4% of the nominal concentrations (Vera-Chang et al., 2018). The embryos from period 1 were exposed from 3 hpf to 15 dpf during early sexual development (Uchida et al., 2004; Tong et al., 2010), whereas the embryos from period 2 were exposed to the same FLX concentrations beginning at 15 dpf and ending at 42 dpf during the period that encompasses the period of sex differentiation in ZF (late sexual development) (Orban et al., 2009).

Embryos from both exposure groups were reared as previously described (Vera-Chang et al., 2018). All experiments were conducted under a protocol approved by the University of Ottawa Animal Care Protocol Review Committee and undertaken in accordance with institutional animal care guidelines adhering to those of the Canadian Council on Animal Care.

### Acute Stress Response

Six-month-old females and males from both FLX exposures and the CTR were subjected to a standardized net handling stressor (Ramsay et al., 2009) as previously described (Vera-Chang et al., 2018). Briefly, 1 week prior to the stressor, the fish were transferred to the testing room and allowed to acclimate to the experimental tanks. On the day of the stress test, half of the fish from each FLX treatment and sex group were immediately sacrificed (Unstressed group), whereas the other half was subjected as a group to the stressor (Stressed group) (Ramsay et al., 2009) between 09 h15 and 10 h30. The fish were sacrificed by submersion in ice-cold water and subsequently weighed, immediately snap-frozen in liquid nitrogen and stored at −80°C for whole-body cortisol analysis.

### Cortisol Extraction and Quantification

Whole-body cortisol was extracted using a protocol modified from Folch et al. (1957) as previously described (Vera-Chang et al., 2018). Sacrificed fish were frozen rapidly and stored at −80°C for only a few days before extraction. Samples from the same experiment were run together in no more than 3 weeks post-extraction. To avoid any potential degradation, we limited incubation temperatures during sample preparation to not more than 45°C (de Rijke et al., 2014). Individual fish were pulverized and homogenized in CHCl_3_:MeOH (2:1 v/v). After 15 min of incubation at room temperature, 2 M KCl buffered with 5 mM EDTA was added to the homogenate, vortexed and incubated for an additional 20 min. The organic phase was then evaporated to dryness. The lipid extract was reconstituted in ethylene glycol monomethyl ether. The extraction efficiency was calculated to be 87%. Total cortisol concentration was assessed by radioimmunoassay (RIA; MP Biomedicals; Solon, OH, United States) according to the manufacturer’s protocol and using the Wizard2 gamma counter (PerkinElmer; Downers Grove, IL, United States). This RIA kit is highly specific for cortisol, exhibiting only moderate cross-reactivity (∼12%) with 11-deoxycortisol and having a limit of detection of 0.17 μg^⋅^dL^–1^. Diluted extracts, commercial and in-house cortisol standard curves exhibit parallelism (Supplementary Figure S1). The intra- and the inter-assay coefficients of variation (CV) were calculated to be 4–8% and 7–15%, respectively. Cortisol concentrations were not corrected for extraction efficiency.

### Metyrapone Exposure

Adult ZF (8-months-old) of the AB strain bred in-house from a chemically clean population (naïve fish) were exposed in system water to either metyrapone (325 μM; Adooq Bioscience; Burlington, ON, Canada), an 11β-hydroxylase inhibitor, or the DMSO vehicle (0.02% v/v) for 1 week. Effective metyrapone dose and treatment period were estimated using a pilot study to determine levels that inhibited cortisol production in ZF (Supplementary Figure S2). Females and males were placed in separate 5-L glass tanks at a density of 5 fish^⋅^L^–1^ with adequate aeration. The experiment was designed as static renewal, where water was replaced daily 1 h after feeding. Ammonia, nitrate and nitrites where measured, however, no differences were found between groups and all values were within normal ranges. At the end of the 1-week exposure, a group of fish from each FLX treatment and sex were immediately sacrificed to assess unstressed cortisol levels. The remaining fish were subjected to the novel tank diving test (behavioral experiment) prior to being terminally anesthetized, weighed, snap-frozen in liquid nitrogen and stored at −80°C for further whole-body cortisol assessment.

### Behavioral Testing and Analysis

To assess the locomotor and exploratory activities evoked by the habituation response to novelty (Wong et al., 2010; Rosemberg et al., 2011), adult females and males ZF exposed to FLX and metyrapone were subjected to our novel tank diving test (Vera-Chang et al., 2018) adapted from Levin et al. (2007). Briefly, females and males were placed in separate 3-L tanks (16 fish^⋅^tank^–1^) and allowed to acclimatize to the testing room 1 week prior to the experiment. All behavioral testing was performed between 09 h30 and 14 h30 in a trapezoid-shaped tank (Aquatic Habitats) filled with system water. The behavioral activity of each individual fish was recorded for 6 min in fresh system water. Videos were analyzed every 30 frames^⋅^s^–1^ for a total of 10,800 frames using a validated in-house automated tracking (AT) system (Python script) (Vera-Chang et al., 2018). A Python script was also used to calculate the behavioral metrics (Table 1) assessed in this study. Principal component analysis (PCA) was performed on the 10 calculated behavioral metrics obtained from the AT system. One PCA was conducted on the data sets across all animals (females and males). PCA yielded a total of 10 different components (principal components; PC – Supplementary Table S1), however, only PC1 strongly loaded most of the behavioral metrics (Table 1) and explained 42% of the behavioral variance. The two variables that did not robustly contribute to PC1 were maximum speed and total distance traveled. Positive scores are associated with high exploratory and locomotor activities, whereas negative scores are linked to reduced behavioral activities.

**TABLE 1:** Loadings and contributions of the behavioral metrics to PC1.

**Behavioral metrics**	**Description**	**Loadings**	**Contribution (%)**
Latency middle third	Delay before entering the middle third of the tank	–0.225	5.1
Latency top half	Delay before entering the top half of the tank	–0.246	6.0
Latency top third	Delay before entering the top third of the tank	–0.271	7.4
Transitions	Number of times the fish crossed into the top half of the tank	0.406	16.5
Time middle third	Total time spent in the middle third of the tank	0.403	16.3
Time top third	Total time spent in the top third of the tank	0.362	13.1
Distance middle third	Total distance spent in the middle third of the tank	0.413	17.0
Distance top third	Total distance spent in the top third of the tank	0.407	16.6
Total distance	Total distance traveled around the tank	0.115	1.3
Max speed	Maximum speed reached by the fish	–0.084	0.7

### Statistical Analyses

Cortisol levels were expressed as the mean ± SEM, whereas behavioral data were presented in box plots showing the 10 and 90th percentiles. Prior to hypothesis testing, datasets were examined for normality and homogeneity of variance using the Shapiro–Wilk test and Levene’s median test, respectively. A two-way ANOVA was conducted to examine the effects of the stressor and FLX treatments on cortisol levels in each of the exposure periods. Two-way ANOVA was also used to examine the effects of sex and treatments on the stress response of the adult fish from each period. Statistical significance among the groups was determined using Tukey’s *post hoc* test. Box-Cox transformations (Box and Cox, 1964) were applied when data were not normally distributed. Alternatively, two-way ANOVA on ranks (indicated in each graph) was used for data that had non-Gaussian distributions even after undergoing transformations. Two-way ANOVA or two-way ANOVA on ranks (when data did not meet normality) followed by Tukey’s *post hoc* test were conducted to determine significant difference between sex and treatments on the PC1 scores for the behavioral analysis. The level of significance for all tests was set at α < 0.05, and all statistical analyses were performed using SigmaPlot 11.0 (Systat Software, Inc., San Jose, CA, United States).

## Results

### FLX Reduces Cortisol Levels in Adult Female and Male ZF Regardless of the Window of Exposure

**FIGURE 2:**
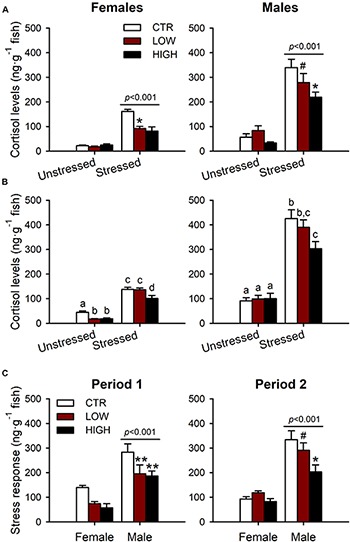
Whole-body cortisol levels (ng^⋅^g^–1^ fish) in adult females and males (6 months of age). **(A)** Adult ZF exposed to FLX from 0 to 15 dpf (period 1, early sexual development) during early development. **(B)** Adult ZF exposed to FLX from 15 to 42 dpf (period 2, late sexual development) during the period of sex differentiation in ZF. **(C)** Stress response (stress-induced cortisol levels – unstressed cortisol levels) in females and males from exposure period 1 and period 2. The two concentrations of FLX used were 0.54 and 54 μg^⋅^L^–1^ denoted as LOW and HIGH, respectively. The stress response was induced using a standardized net handling stress protocol. The data from the three panels are presented as mean ± SEM and analyzed by two-way ANOVA (and on ranks for data on panel **(A)** both graphs, and panel **(C)** period 1), *P* < 0.05. The letters represent statistical difference when interactions are present. *P*-values shown above the bars represent significant difference compared with unstressed [for **(A,B)**] or significant difference between sex [for **(C)**]. The symbols represent significant difference within the FLX treatments: ^∗^*P* < 0.05 compared with CTR, ^∗∗^*P* < 0.005 compared with CTR and ^#^*P* < 0.05 compared with HIGH. *n* = 4–8 per group in females and *n* = 6–10 in males.

To determine if exposure to FLX during two different windows of sexual development induced a disruption of the stress axis similar to that observed in our previous study [27], we examined whole-body cortisol levels following an acute standardized net handling stressor in adult female and male ZF. Whole-body cortisol levels were significantly elevated by the acute stressor in the adult females (*F*_1__,__34_ = 110.153, *P* < 0.001) and males (*F*_1__,__37_ = 119.887, *P* < 0.001) from the CTR and FLX treatments following exposure period 1 (Figure 2A). However, FLX treatment significantly reduced the whole-body cortisol levels in the adult females (40 and 42% reductions in the LOW and HIGH treatments, respectively; *F*_2__,__34_ = 4.404, *P* = 0.020) and the adult males (8 and 36% reductions; *F*_2__,__37_ = 4.596, *P* = 0.016; Figure 2A) when compared with their matched CTR. No interactions between FLX treatment and the stressor were found in the adult females (*F*_2__,__34_ = 2.627, *P* = 0.087) and males (*F*_2__,__37_ = 1.617, *P* = 0.212) following exposure period 1 (Figure 2A).

The acute stressor was also effective at eliciting a stress response in the adult females (*F*_1__,__30_ = 307.812, *P* < 0.001) and males (*F*_1__,__41_ = 169.485, *P* < 0.001) from the CTR and FLX treatments following exposure period 2 (Figure 2B). However, similar to the treated-fish from period 1, treatments with FLX during exposure period 2 (Figure 2B) led to an attenuation in the stress-induced cortisol levels in the adult fish, but this impairment was less pronounced and only statistically significant in females (15 and 34% reductions in the LOW and HIGH treatment, respectively; *F*_2__,__30_ = 11.348, *P* < 0.001) but not in males (*F*_2__,__41_ = 2.625, *P* = 0.085). In contrast to period 1, there was a statistically significant interaction between FLX treatments and the stressor in the adult females (*F*_2__,__30_ = 4.602, *P* = 0.018) and males (*F*_2__,__41_ = 3.366, *P* = 0.044) following exposure period 2. More specifically, the LOW and HIGH FLX treatments following exposure period 2 reduced the whole-body cortisol levels in the adult females at resting conditions (unstressed), whereas upon the stressor, only the adult females from the HIGH FLX treatment experienced this whole-body cortisol reduction. Conversely, the unstressed cortisol levels of the FLX treated adult males were not affected following exposure period 2. However, exposure to the HIGH FLX treatment during period 2 resulted in a reduction of the whole-body cortisol levels in the adult males following the acute stressor.

Furthermore, to investigate whether FLX exposure results in sex-specific effects on the disruption of the stress axis, we compared the stress response of the adult females with the adult males across treatments following FLX exposure during period 1 and period 2 (Figure 2C). The stress response was calculated as the arithmetic difference between the stress-induced cortisol levels and the unstressed cortisol levels. Simple main effects analysis showed that the stress response was significantly higher in males compared with females from exposure period 1 (*F*_1__,__34_ = 56.707, *P* < 0.001) and exposure period 2 (*F*_1__,__36_ = 75.234, *P* < 0.001) regardless of the treatment (Figure 2C). We also found that FLX exposure during period 1 (*F*_2__,__34_ = 9.002, *P* < 0.001) and period 2 (*F*_2__,__36_ = 4.109, *P* = 0.025) significantly reduced the stress response of the adult fish (Figure 2C). However, no interactions were found between sex and FLX treatments following exposure during period 1 (*F*_2__,__34_ = 0.348, *P* = 0.709) or period 2 (*F*_2__,__36_ = 2.132, *P* = 0.133; Figure 2C).

### FLX Exposure During Sexual Development Results in Sex- and Window of Exposure-Specific Effects of the Exploratory Behavior in Adult ZF

**FIGURE 3:**
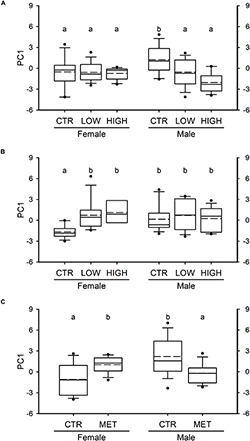
Behavioral response following the novel tank diving test in adult ZF (6 months of age). **(A)** PC1 of females and males exposed to FLX (0.54 and 54 μg^⋅^L^–1^, LOW and HIGH, respectively) from 0 to 15 dpf (period 1) during early sexual development. **(B)** PC1 of females and males exposed to FLX (0.54 and 54 μg^⋅^L^–1^, LOW and HIGH, respectively) from 15 to 42 dpf (period 2) during the period of sex differentiation (late sexual development) in ZF. **(C)** The behavioral response of females and males exposed to metyrapone (MET; 325 μM) as adults for 1 week. In all panels, PC1 represents the exploratory behavior of the fish, where positive scores are associated with high exploratory activity, and negative scores are linked to reduced behavior. Data were analyzed using two-way ANOVA on ranks [and two-way ANOVA for panel **(A)**]. Data are presented as box plots showing the median (solid line), the mean (dashed line), the interquartile range (box) and the whiskers embracing data within the 10 and 90th percentiles; all data outside the range of the whiskers are presented as individual data points. *n* = 12–14 fish per group in panel **(A)**, *n* = 8–13 per group in panel **(B)** and *n* = 15 per group in panel **(C)**. The letters in all panels represent statistical difference when interactions are present.

To examine whether exposure to FLX during different periods of sexual development induced sex- and time-dependent effects in the exploratory behavior in the adults, we subjected the fish to the novel tank diving test. The behavioral response of the adult fish was significantly affected by FLX treatments following exposure period 1 (*F*_2__,__70_ = 6.802, *P* = 0.002; Figure 3A) as well as exposure period 2 (*F*_2__,__66_ = 7.264, *P* = 0.001; Figure 3B). More specifically, FLX treatments induced a reduction in the exploratory behavior following exposure period 1, whereas the FLX-treated adult fish from exposure period 2 exhibited an increase in their behavior when compared with their matched CTR. Sex alone was not a contributor to the effects observed in the behavioral response of the adult fish from exposure period 1 (*F*_1__,__70_ = 0.153, *P* = 0.697; Figure 3A) or exposure period 2 (*F*_1__,__66_ = 0.667, *P* = 0.417; Figure 3B). However, there was a statistically significant interaction between sex and FLX treatments on the behavioral response of the adult fish from exposure period 1 (*F*_2__,__70_ = 5.265, *P* = 0.007; Figure 3A) and exposure period 2 (*F*_2__,__66_ = 5.269, *P* = 0.008; Figure 3B); the behavioral response of the males was only affected following FLX treatment during exposure period 1 (a reduction was observed; Figure 3A) whereas the behavior of the females was only affected following FLX treatment during exposure period 2 (an increase was observed; Figure 3B).

### Recapitulation of the Behavioral Response Observed in the FLX-Treated Fish by Chemically Reducing Cortisol Levels in Naïve Fish

Chemical reduction of cortisol levels by exposure to metyrapone (Supplementary Figure S3), a steroid 11β-hydroxylase inhibitor, in naïve adult female and male ZF recapitulated the sex- and window of exposure-specific behavioral responses to the novel tank diving test observed in our FLX-treated fish. Neither metyrapone treatment (*F*_1__,__56_ = 0.022, *P* = 0.883; Figure 3C) nor sex (*F*_1__,__56_ = 1.184, *P* = 0.281; Figure 3C) had an effect on the exploratory activity of the adult fish. However, the main effect of sex significantly interacted with the metyrapone treatment (*F*_1__,__56_ = 16.188, *P* < 0.001; Figure 3C). More specifically, metyrapone treatment increased the behavioral response in the adult females whereas in the adult males, metyrapone reduced their response to the novel tank test.

## Discussion

Here, we have uncovered that exposure to the antidepressant FLX during two different windows of sexual development in ZF disrupted their adult stress axis in females and males at 6 months of age. This disruption was manifested by a decrease in whole-body total cortisol levels. Similar magnitude of impairment in total cortisol levels was induced following FLX exposure during both early sexual development (period 1; 0–15 dpf), where the juvenile ovary starts to differentiate regardless of the sexual genotype, and late sexual development (period 2; 15–42 dpf) during gonadal differentiation. These findings on the disruption of the stress axis by FLX are in accordance with our previous study where exposure from 0 to 6 dpf reduced the whole-body cortisol levels following an acute stressor in adult female and male ZF (Vera-Chang et al., 2018). In our earlier study, the FLX-treated females displayed 13–30% reduction in the stress-induced whole-body cortisol levels compared with the CTR females, whereas the FLX-treated males exhibited 38–57% reduction relative to the CTR males (Vera-Chang et al., 2018). In the current study, however, there was a similar magnitude of disruption in the stress-induced whole-body cortisol levels in both female and male FLX-treated ZF. These differences between our two studies in the magnitude of cortisol disruption induced by FLX may be attributable to the duration of the exposure; in our previous study, the exposure was conducted for 6 days whereas in our present study, the FLX exposure during early sexual development was carried out for 15 days. Thus, it is important to consider different durations of exposure when studying the effects of endocrine disruptors such as FLX.

Another important finding is the sex differences in the stress response following both periods of exposure regardless of the treatment. The stress response of the females from all treatments were 28–49% of that of males. Sex differences in HPA reactivity have been extensively studied in mammals, especially humans and rodents (Uhart et al., 2006; Herman et al., 2016). This sexually dimorphic stress response is partly the reflection of the organizational and activational effects of differential sex steroid levels in the two sexes (Frye et al., 2012; Rosenfeld et al., 2017). However, it is important to highlight that these sex-specific differences in the magnitude and duration of the HPA response to a stressor in humans and rodents vary according to the age of the individual, estrous cycle, and type and severity of the stressor (Kudielka and Kirschbaum, 2005; DeSantis et al., 2011). In ZF, evidence for sexual dimorphism in the stress response is equivocal (Filby et al., 2010; Fuzzen et al., 2011; Oswald et al., 2012; Félix et al., 2013; Zhang et al., 2015). However, several studies reporting these sex differences in cortisol levels are in accordance with our findings, where females exhibit a lower stress response to a stressor than males (Oswald et al., 2012; Zhang et al., 2015). Sex-dependent differences in the HPI axis have also been documented in other teleosts such as yellow perch (*Perca flavescens*), rainbow trout (*Oncorhynchus mykiss*) and sockeye salmon (*Oncorhynchus nerka*) (Girard et al., 1998; Pottinger and Carrucj, 2000; Kaoru et al., 2001; Øverli et al., 2006). In these teleosts, females displayed higher stress-induced cortisol levels than males. However, similar to mammals, the sex-specific cortisol differences vary according to the sexual maturity of the fish and/or their reproductive periods. In our present study, we demonstrated that FLX exposure during both windows of sexual development induced a disruption of whole-body total cortisol levels in adult females and males. However, it remains to be determined whether the enduring effects of FLX on the stress axis following exposure period 1 (from 0 to 15 dpf) results from the same mechanism(s) of disruption as exposure period 2 (from 15 to 42 dpf).

The behavioral response to the novel tank diving test of the FLX-exposed adult fish was found to differ according to sex and window of exposure. Our findings revealed that females are more sensitive to the effects of FLX during the period of sex differentiation (period 2), since they displayed a significant increase in their exploratory behavior compared with their matched CTR females. In contrast, adult males exhibited reduced activity following the FLX exposure period 1, suggesting that the males are more sensitive to the long-lasting disruptive effects of FLX during their early development. Sex- and age-dependent behavioral alterations induced by FLX have also been observed in rodents. For instance, young adult and middle-aged female rats were found to be more sensitive than male rats to the FLX effects following the forced swim test (Fernandez-Guasti et al., 2017). These females responded to 5 and 10 mg^⋅^kg^–1^ FLX by decreasing their immobility, whereas male rats only responded to 10 mg^⋅^kg^–1^. In the same study, the authors reported that senescent female rats treated with 10 mg^⋅^kg^–1^ FLX also exhibited a reduction in immobility. However, this effect was completely absent in senescent male rats (Fernandez-Guasti et al., 2017). Another study focusing on the effects of FLX on fear responses after conditioned fear extinction, demonstrated that acute administration (one time) to FLX increased fear responses equally in female and male rats (Lebron-Milad et al., 2013). However, chronic FLX administration (14-day treatment) reduced fear responses during extinction learning and extinction recall in female rats only. The authors also reported that these effects of chronic FLX treatment on the female rats were modulated by gonadal steroid levels; the reduction in fear responses was more pronounced in females during their metestrus/diestrus cycle (low estrogen phase) (Lebron-Milad et al., 2013). In mammals, it is well-know that behavioral responses to different stimuli vary according to the sex and the age of the organism when the stimulus and/or stressor is perceived (Bale and Epperson, 2015). The main studied mechanism responsible for establishing sex differences derives from the organizational/activational hypothesis of sexual differentiation (McCarthy et al., 2009). This hypothesis stipulates that sex differences in behavioral responses are likely the reflection of the differential timing of neural maturation and organization in females and males, which are mainly controlled by the sex-dependent steroid levels (Frye et al., 2012). In ZF, the period of sexual development is highly sensitive to sex steroids as sex determination, in addition to the differentiation and development of their gonads, are driven primarily by androgens and estrogens (Luzio et al., 2015). Moreover, sexual development studies in ZF indicate that the window of vulnerability differs for androgens and estrogens; ZF are more sensitive to androgens during early sexual development and to estrogens at the end of their sexual developmental period (Andersen et al., 2003; Uchida et al., 2004). Therefore, it is possible that the sex differences in the behavioral response observed in our FLX-treated fish are related to the sex-dependent steroid levels. Additionally, sex differences in serotonin production in the brain (Dahlbom et al., 2012) could also contribute. However, further research needs to be conducted to validate this hypothesis. Our experimental design is such that we targeted critical exposure periods that covered specific developmental stages rather than a specific duration. It is important to note that exposure period 1 (15 days) about half the length of exposure period 2 (27 days). There is thus a possibility that some of the difference observed could be confounded by exposure duration.

The most striking finding of this study was the unexpected difference in the behavioral response to novelty in the females compared with that of the males as a result of the FLX-induced low cortisol levels. These results were supported by the behavioral response of naïve females and males following a chemical inhibition of cortisol synthesis with metyrapone. The metyrapone-exposed adult females were more active during the novel tank diving test, whereas the behavioral response of the metyrapone-treated adult males was significantly reduced relative to their matched CTR fish. Sex differences in behaviors in ZF including boldness, cocaine withdrawal, and shoaling preferences and selection have been previously reported (Ruhl and McRobert, 2005; Engeszer et al., 2008; López Patiño et al., 2008; Oswald et al., 2012). It should also be noted that a 1-week exposure to metyrapone in adult ZF can recapitulate the behavioral responses observed in the younger FLX-treated fish suggests that the sex-specific behavioral effects of the FLX treatment may be somewhat independent of the age and stage of development. To our knowledge, this is the first study to report on sexual dimorphism in the exploratory activity of adult ZF as a result of impaired cortisol levels. These sex differences may be attributable to sex-specific coping mechanisms and/or to the sexual dimorphism of brain organization, possibilities that require experimental validation.

## Conclusion

This study provides evidence that FLX has the ability to disrupt both the stress axis and behavioral activity in adult female and male ZF, effects that are dependent on the window of exposure and the sex of the exposed individual. These findings highlight the importance of sex differences and timing in response to antidepressants administration and/or exposure. Considering the continuous discharges of FLX into the aquatic environment, knowledge of the sex-specific windows of susceptibility to antidepressant effects and the importance of exposure to these chemicals may advance our understanding of the potential effects of FLX on the fitness, survival and population dynamics of aquatic organisms that are being exposed (Yang et al., 2017; Comber et al., 2018). In addition, understanding the role of sex in the effects induced by FLX and the window of sensitivity of females compared with that of males, may help us with our efforts to better determine the best antidepressant therapeutic strategy for each sex with the aim of minimizing any unintended effects of the antidepressant on the treated patient. It is also important to highlight that FLX is the first-line of pharmacological treatment in women during their pregnancy and post-partum period as well as in adolescents (Latendresse et al., 2017; Locher et al., 2017; Man et al., 2017; Morkem et al., 2017; Sarginson et al., 2017). Since early development including fetal, infancy and adolescence, is highly plastic, our study and others like it are necessary to better understand the possible enduring effects of FLX on adults exposed during highly plastic periods of development.

## Data Availability Statement

All datasets generated for this study are included in the manuscript/Supplementary Files.

## Ethics Statement

All experiments were conducted under a protocol approved by the University of Ottawa Animal Care Protocol Review Committee and undertaken in accordance with institutional animal care guidelines adhering to those of the Canadian Council on Animal Care.

## Author Contributions

MV-C designed the study, developed the methodology, conducted the experiments and analyses, and prepared the manuscript. AS-J wrote the Python scripts for the behavioral analyses. CL measured FLX concentrations in the water. TM and VT helped with the design of the study and manuscript editing.

## Conflict of Interest

The authors declare that the research was conducted in the absence of any commercial or financial relationships that could be construed as a potential conflict of interest.
