# Supplementary material for: Fluoxetine Exposure During Sexual Development Disrupts the Stress Axis and Results in Sex- and Time- Dependent Effects on the Exploratory Behavior in Adult Zebrafish Danio rerio
Source: Front Neurosci. 2019 Sep 19;13:1015. doi: 10.3389/fnins.2019.01015 (PMC6761223; doi:10.3389/fnins.2019.01015)
Supplement: Supplementary file 1 [file Data_Sheet_1.docx]

Supplementary Material

# Supplementary Figures and Tables

## Supplementary Figures

**Supplementary Figure 1.** Validation of the I^125^-RIA (MP Biomedicals) with our cortisol extraction matrix. **(A)** Regression plot of the standards from the kit and our homemade standards diluted in EGME (solvent used in our samples). **(B)** Parallelism of the diluted whole-body extracts with the standard curves. **(C)** Regression plot of the standards alone and standards diluted with the cortisol extracted matrix shows parallelism of both curves.

**Supplementary Figure 2.** Whole-body cortisol levels (ng·g^−1^ fish) in adult ZF following a week of metyrapone exposure. Metyrapone-exposed fish were either killed right after exposure (Unstressed group) or intraperitoneal (i.p.) injected with saline solution (10 μL·g^−1^ body weight – Saline group) or i.p. injected with adrenocorticotropic hormone (0.0625 IU·g^−1^ body weight – ACTH group). The ACTH concentration was estimated by conducting a prior dose–response experiment from which the minimal stimulatory ACTH concentration that triggered the maximum synthesis of cortisol was selected. Before the i.p. injection, fish were anesthetized in a 60 μM ethyl 3-aminobenzoate methane sulfonate salt solution, weighed, and immediately placed on a sponge saturated with cold water. The injection procedure was conducted in such a way as to guarantee that the animal did not spend more than 20 s out of water. After the injection, the animals were individually placed in a tank with system water, and 30 min following their recovery from the anesthesia, the fish were killed. All of the animals recovered in less than 1 min, and no mortalities occurred from the injection. Injections were performed between 09 h30 and 12 h30.

**Supplementary Figure 3.** A week metyrapone (MET) exposure inhibited the total cortisol production in naïve adult **(A)** female and **(B)** male ZF subjected to the novel tank diving test (NTT). STR-NTT, stressed levels following the NTT; n = 4 to 7 and 16 fish for basal and novel-tank diving test groups, respectively.

## Supplementary Tables

**Supplementary Table 1. Loadings and contributions of the 10 different PCs and behavioral metrics of the behavioral test after PCA analysis**

|  | **PC1** | **PC2** | **PC3** | **PC4** | **PC5** | **PC6** | **PC7** | **PC8** | **PC9** | **PC10** |
| --- | --- | --- | --- | --- | --- | --- | --- | --- | --- | --- |
| **PC weights (%)** | 42.4 | 20.9 | 16.5 | 7.1 | 5.6 | 3.7 | 1.8 | 1.4 | 0.5 | 0.1 |
| Latency middle third | 5.06 | 23.40 | 3.58 | 0.65 | 0.85 | 58.40 | 0.71 | 6.85 | 0.44 | 0.05 |
| Latency top half | 6.03 | 29.80 | 1.30 | 0.12 | 0.08 | 2.95 | 8.72 | 50.70 | 0.19 | 0.11 |
| Latency top third | 7.35 | 24.23 | 0.03 | 0.02 | 0.21 | 35.69 | 4.03 | 28.35 | 0.00 | 0.09 |
| Transitions | 16.49 | 5.85 | 2.31 | 5.28 | 0.91 | 0.21 | 42.87 | 8.91 | 10.57 | 6.59 |
| Time middle third | 16.28 | 5.46 | 0.08 | 14.02 | 4.20 | 0.00 | 34.50 | 3.25 | 1.42 | 20.80 |
| Time top third | 13.13 | 0.30 | 14.70 | 23.76 | 0.89 | 0.29 | 1.52 | 0.02 | 34.44 | 10.96 |
| Distance middle third | 17.05 | 6.50 | 3.79 | 9.07 | 0.35 | 0.00 | 0.05 | 0.29 | 10.56 | 52.34 |
| Distance top third | 16.60 | 0.98 | 3.97 | 24.78 | 1.23 | 1.15 | 1.77 | 0.27 | 40.62 | 8.64 |
| Total distance | 1.31 | 3.40 | 32.48 | 14.48 | 39.17 | 1.30 | 5.74 | 1.34 | 0.38 | 0.39 |
| Max Speed | 0.71 | 0.09 | 37.76 | 7.82 | 52.10 | 0.00 | 0.09 | 0.03 | 1.38 | 0.03 |
